# Two Sides of the Same Coin or Two Different Currencies? Representations of Happiness and Unhappiness among Finnish Women

**DOI:** 10.1007/s12124-020-09579-4

**Published:** 2020-10-16

**Authors:** Jennifer De Paola, Wolfgang Wagner, Anna-Maija Pirttilä-Backman, Josetta Lehtonen

**Affiliations:** 1 Faculty of Social Sciences, Social Psychology, University of Helsinki, P.O. Box 54, 00014 Helsinki, Finland; 2 University of Tartu, Tartu, Estonia

**Keywords:** Happiness, Unhappiness, Social representations, Word associations, Women

## Abstract

This paper presents results from a study exploring representations of “happiness” and “unhappiness.” Word associations with these concepts were produced by 16–18 and 29–34-year-old women from Finland, the country that the United Nation’s World Happiness Report has ranked the “happiest” in the world. Correspondence Analysis (CA) and Hierarchical Cluster Analysis show that participants in both age groups share three clusters of words associated with “happiness”: *Tangible happiness*, *Affective happiness* and *Serene happiness*. We noted more differences in the associations with “unhappiness,” for which the two groups share only two clusters: *Loss* and *Everyday problems.* A distinct third cluster, *Affective unhappiness*, emerged for the younger women, whereas older women’s associations are further differentiated into a more complex structure, including two more clusters: *Dejection* and *Apprehension*. Additionally, CA shows that in both age groups, self-reported happiness levels do not discriminate which words are associated with happiness and unhappiness. Finally, qualitative content analysis of a questionnaire item investigating how to reach complete happiness suggested that there are three recurring answer types: happiness can be improved through external changes, internal changes, or not at all because complete/permanent happiness does not exist. The study provides a methodological design which, unlike most happiness studies, allows participants the freedom to bring up the meaning of happiness and unhappiness. Thus, the study constitutes a contribution to a more nuanced understanding of happiness.

“All happy families are alike; each unhappy family is unhappy in its own way.”

—Leo Tolstoy, *Anna Karenina*

Against stereotypical expectations of Finns as emotionally introverted people dealing with long and dark winters brought by the boreal climate, for the past three years the World Happiness Report (WHR) has ranked Finland as the happiest country in the world (Sachs et al. 2018; Helliwell et al. 2019, 2020). When asked how it feels to live in the “happiest country in the world,” an 18-year-old Finnish girl mused: “*kind of a joke if you think about the grey weather and the people taking the stairs to avoid talking to their neighbor, but makes sense when you think about our social welfare really working and things like free education*.” With her brief answer, this young Finnish woman acknowledged the multifaceted nature of happiness, venturing a guess at the “type” of happiness studied by the WHR while at the same time hinting at possible other ways to “understand” happiness, which were ignored by the survey and caused the result to be perceived, at least partly, as a “joke.”

## Aspects of happiness

The results of the World Happiness Report usually attract a great deal of publicity but little analysis of what happiness is, what it means to people, and how to best operationalize it. The WHR reaches its conclusions by asking inhabitants in 156 countries to rank their current lives on a scale of 0–10, with 0 referring to the worst possible life and 10 to the best possible life. However, when considering the positive and negative affect measures in the same study—namely, the average frequency of happiness, laughter and enjoyment and the average frequency of worry, sadness and anger experienced during the previous day—Finland would rank 41st on the positive side and 146th on the negative. Thus, it can be called into question how close the concepts of *best possible life* and *happiness*—or other usually positively evaluated affects—really are in people’s minds.

Combining this observation with the general trend in scientific literature to use happiness, well-being and other related terms in varying and often even interchangeable ways, this study aims to achieve a better understanding of the social representations of happiness and unhappiness. We propose that a more comprehensive understanding of these constructs can be gained by exploring them conjointly.

As biological beings, humans from all over the world prefer what is desirable over the undesirable and the pleasant over the unpleasant (Michalos 1991; Veenhoven 1991). Positive feelings have evolved because they “make biological sense,” as they play a paramount role in increasing the survival of our genes (Grinde 2012). Thus, the pursuance of pleasure (and avoidance of pain) is hardwired in the evolutionary process.

Gradually, the endeavor for happiness has been integrated into our institutions. The mention of the “pursuit of happiness” in the United States’ Declaration of Independence in 1776, defined as one of the unalienable rights given to all humans by their creator, engendered a process of incorporating the genetic tendency to seek “happiness” into an institutionalized moral imperative, making happiness one of Western society’s most cherished goals (Veenhoven 1994). More recently, leading politicians have included the increase of happiness in their political agendas (The Happiness Effect 2011).

In the current scientific literature, it is possible to distinguish two ways of defining happiness, a split that can be traced back to the Ancient Greek philosophical distinction between *hedonism*, or happiness as a quest for pleasure, and *eudemonia*, or happiness as a quest for the meaning of life. The former is reflected in the substantial number of empirical studies approaching the topic of happiness from the perspective of the Subjective Well Being model (SWB) proposed by Diener (1984). The SWB construct includes an affective component named hedonic well-being (*pleasure* vs. *pain*) on the one hand and the cognitive judgement of one’s life understood in terms of *life satisfaction* on the other. Other researchers (e.g., Seligman 2002) refer to happiness in terms of *eudemonia*, defining happiness as the extent to which we perceive life to be meaningful and worth living. Eudomania is not a mere sum of positive affects, nor does it correspond to being satisfied with life; in fact, both positive and negative emotions are required in order to experience personal growth. The emphasis is placed on *flourishing* and elevating oneself rather than being satisfied.

As Salmela (2008) has put it, happiness is “*both a very philosophical and a very empirical matter – as well as being both research-oriented and everyday life-oriented*” (p. 4). And yet, except for a limited number of studies (e.g., Delle Fave et al. 2011, 2016; Oishi et al. 2013) we have very little knowledge on how philosophically and empirically derived conceptualizations of eudemonic and hedonic happiness map onto the understanding of this concept among the general public. Phenomena related to happiness, ranging from its antecedents to its outcomes and levels, have received more empirical attention compared to the actual *meaning* that people attribute to happiness (Carlquist et al. 2017). For example, in her quest to gain more knowledge about Finnish people’s happiness, Pessi (2008) asked 1051 people to rate how important the elements included in a ready-made list were (on a scale from 0 to 5), producing interesting results showing the most important elements in the happiness of Finns: *human relationships*, *health* and *stable income*. At the same time, such research designs have the tendency to reduce the focus of happiness to its antecedents, assuming that the participants agree with the study’s assumptions of what “being happy” means, thus precluding any insight into the participants’ own understandings of the concept.

Empirical studies following a different approach to exploring everyday understandings of happiness have underlined both commonalities and cultural specificities in how this concept is conceptualized. Kövecses (2008), for example, starting from the study of conventionalized linguistic expressions (e.g., metaphors and metonyms) relating to happiness in the English language proposes that on a *general* level, folk theories on happiness rely on a tripartite prototype of the idea: happiness as an “immediate response,” happiness as a “value” and happiness as “being glad.” According to Kövecses, people attribute different meanings to happiness based on the degree of intensity of certain emotional responses; happiness as an “immediate response” is intense, hardly noticeable as a “value” and mild as “being glad.”

In contrast, in his cross-cultural studies on the social representations of happiness in Italy and Chile, Rodríguez-Araneda (2013) has focused on the *cultural specificity* of everyday understandings of happiness. The results of that study showed that, on the one hand, in specific contexts the social representations of happiness were organized around common nuclei (i.e., positive emotionality, accomplishment and feeling good). On the other hand, there were different ways of understanding happiness: the Chilean group identified happiness with fullness, well-being and enjoying life, while the Italian group found more importance in interpersonal affections.

Of direct relevance to the present research is the study by Shin et al. (2018) were participants from Korea and US were asked to name three words that come to mind is association with term “happiness”. Results showed that the most frequently endorsed words were “family” among Koreans and “smile” among Americans. In addition, in both cultures participants who mentioned more social words (e.g. “family” and “friends”) also reported to be more satisfied with their lives.

Such results are intriguing because they challenge the idea of a universal definition of happiness, emphasizing the role of cultural differences as well as the need to explore different ways of understanding the same word.

### _What Is the Opposite of Happiness?_

Most studies on happiness in general and those focusing on the *meaning* of happiness in particular start with the premise that the nature of this concept is resistant to any definition. Contributing to the difficulties in finding a unanimously accepted definition of happiness is the fact that in many languages this concept does not have a clear opposite. In the English language, for example, the term used most often to indicate the opposite of happiness is ‘unhappiness.’ The prefix ‘un-,’ meaning ‘not,’ is used as an English formative to give negative or opposite force in adjectives and their derivative adverbs and nouns.

This is problematic in the case of the English language, as happiness/unhappiness do not form either binary opposites, such as ‘bad/good,’ or continuous opposites, as in the case of ‘light/darkness’ (Pawelski 2013). Yet, the issue is perhaps even more complicated when one looks at other languages where the opposite of happiness cannot be defined by the use of an equivalent of the ‘un-’ prefix. This is the case with the Finnish language, where the word *onnellisuus* (‘happiness’) cannot be turned into its opposite by adding a prefix. In spite of this linguistic challenge, and the much-debated first-place position of Finland in the WHR notwithstanding, the question of binaries is of great significance when regarding emic approaches to the processes of sense-making of happiness and unhappiness. For example, the latest available statistics regarding mental health in the Finnish population show that the level of depression in the general population is high, and the incidence of depression in women is higher than for men across all age groups (Murto et al. 2018). How can the same nation rank very high in terms of both “happiness” and depression?

One of the most obvious answers resides in the shortcomings that come with relying on scaled instruments for measuring conceptually unclear or “fuzzy” concepts like happiness, on the one hand, and the assumption that depression rates can be regarded as a symmetrical or continuous opposite of happiness on the other hand.

In the scientific literature on happiness, this asymmetry between happiness and its opposite was already reflected in both of the major happiness paradigms presented earlier. The SWB model (Diener 1984), which defines happiness as frequent positive affect, infrequent negative affect and cognitive evaluations such as life satisfaction, includes a separate dimension for positive and negative emotions, implying that those are not mutually exclusive and stem from two different realms rather than being two (perfectly) symmetrical opposites. Similarly, Seligman (2002) argues that happiness needs to be understood as something more than the simple absence of negative emotions, which would not automatically put us in the position of flourishing and experiencing personal growth but rather in an emotionally neutral state. In addition, empirical examples show us that it is possible to experience feeling sad and happy at the same time (Larsen et al. 2001).

In their study, Uchida and Kitayama (2009) compared descriptions of happiness and unhappiness spontaneously generated by U.S. and Japanese participants. The study indicated significant cross-cultural similarities and differences for folk models of both happiness and unhappiness. In terms of differences, Americans associated happiness with personal achievement, whereas Japanese associated happiness with social harmony. In addition, the participants’ descriptions of unhappiness included different elements of copying behaviour: Americans described unhappiness in terms of external circumstances, whereas Japanese participants associated unhappiness with the concept of self-improvement. These findings showed that although there seems to be a meaningful correspondence between the two concepts, unhappiness can hardly be considered as a perfect ‘mirror image’ of happiness. A possible explanation for this asymmetry resides in the different degree of desirability characterizing happiness and unhappiness, which is reflected in the way cultures place major value on the concept of happiness. In addition, folk models of unhappiness are shaped by the cultural copying mechanism adopted to avoid ‘being unhappy’, which are not needed in the conceptualization of happiness (Uchida and Kitayama 2009).

We propose that in order to accurately comprehend the meaning-making processes of happiness when confronted with the paradox of high rankings of both happiness and depression rates, as in the Finnish context, special emphasis should be placed on exploring the concept of happiness without imposing ready-made definitions on the construct itself and, by extension, its opposite.

Additionally, in light of the disparity between the mental well-being of men and women (Murto et al. 2018), we suggest that exploring everyday understanding among women is a fruitful starting point. In the present study, we specifically focus on women belonging to two particular age groups: 16–18 and 29–34-year-old women. We have a twofold rationale for selecting these age groups. First, we are striving to represent the two different generations known as *Generation Y*, whose members (often referred to as “Millennials”) are born between the 1980s and 1990s, and *Generation Z*, whose members were born in 1995–2015. A comparison between the two generation is compelling for several reasons, but mainly because, having experienced social media and the advent of the internet at different stages of life, these two cohorts privilege different modes of communication and socialization, values, motivations, and attitudes (Wiedmer 2015). Secondly, we believe these age ranges represent two of the most crucial life phases of women. Adolescence is regarded as an undoubtedly critical phase in the process of identity-shaping for both genders (Erikson 1994), as it involves reevaluating values and the salient concepts formed during childhood (Magen 1996) and undertaking a number of future-oriented decisions which contribute to shaping later adult life (Nurmi 1991). Based on statistics released by the Finnish National Institute of Health and Welfare (Heino et al. 2017) and Official Statistics Finland (2017), we have reason to believe that the second age group (29–34 year olds) constitutes another crucial phase in the life of young female adults. Two life milestones considered important by many young adults tend to be achieved during these years: the birth of a first child (Finnish national mean age of a primipara is 29, while the mean age of all parturients is 30) and the official change in the relationship status (first-time marriage rates are highest in the 30–34 age group among women).

### Happiness and Unhappiness as Forms of Social Knowledge

While there is no formal agreement on how happiness should be defined and operationalized, most scholars would agree on the societal saliency of this construct. Library shelves are overflowing with self-help books urging readers to follow the “right steps” toward happiness, dozens of mobile device applications promise users to assist them in tracking their happiness and implementing mindfulness in their daily routines, and most parents would probably declare that “they want their children to be happy” when discussing educational goals.

Without necessarily pondering the meaning of happiness, laypeople and happiness researchers alike have been mainly concerned with how to *increase* happiness levels. For example, according to Ojanen (2008), when asked how happiness can be improved, people tend to mention recurring factors, such as “positive relationships with loved ones, different types of success, hobbies, pets, travelling, nature and small everyday matters” (p. 46). Other scholars (Boehm and Lyubomirsky 2009) have empirically proven the efficacy of various practices (e.g., practicing random acts of kindness, expressing gratitude) in relation to happiness levels. What is more, the concept of happiness has also penetrated political discourse, as shown, for example, with former UK Prime Minister David Cameron undertaking to inquire about citizens’ happiness and what the government could do to promote it (BBC News 2010).

When a topic such as happiness acquires such incontestable societal saliency, individuals and groups will rely on (a system of) common understandings to communicate about and make sense of the object in question. Thus, we propose that the local understandings of happiness in countries and cultures form (a) social representation(s). That is, making sense of happiness is a kind of commonsense that is elaborated, shared and continually renegotiated in everyday communication occurring between individuals and groups, as well as through media and social media platforms.

Social Representations Theory (SRT) acknowledges the coexistence of “scientific” and “commonsense” (or lay theories) and their functions in different fields of knowledge (Moscovici 1994). In this way, it is a fitting theoretical and methodological tool for exploring people’s everyday thinking and sense-making of an array of societally salient topics.

Originally conceived as a theoretical approach to underline the way in which people deal with abstract, threatening, unfamiliar concepts that penetrate public discourse (Moscovici and Vignaux 1994), SRT has also been profitably employed in the study of such societally salient concepts as mental illness (Jodelet 1991), human rights (e.g., Staerklé et al. 1998; Pirttilä-Backman et al. 2017), history and collective memories (e.g., Hakoköngäs and Sakki 2016; Liu and Hilton 2005) and trust (e.g., Pirttilä-Backman et al. 2017), deepening our knowledge about the way in which laypeople understand these phenomena.

Social representations serve two functions: guiding people in understanding and making sense of the world around them and equipping people with a system of common reality, which makes communication possible (Moscovici 1973). These functions are carried out through the processes of anchoring, which entails locating the strange or foreign within the familiar, and objectification, where something abstract is transformed into something concrete. Anchoring and objectification are highly intertwined, dynamic processes which take place during encounters and interactions (Moscovici and Vignaux 1994). Once the unfamiliar is made familiar through anchoring and objectification, the social representation is “naturalized”: ideas that were new and/or abstract enter our everyday language and become part of our social and cultural reality (Sakki and Menard 2014).

The basic premises of our work is that because everyday thinking is by nature antinomic (Marková 2000; Moscovici and Vignaux 1994; Needham 1973), lay understandings of happiness can be, to a certain extent, shaped by the lay understandings of unhappiness (and vice versa). The concept of themata—oppositional thinking—is a central notion within SRT. As noted by these researchers thinking in polarities (right/left, dark/light, good/bad, long/short etc) is adopted across different human societies because it helps us define different concepts. For example, we are able to make sense of the concept of length by defining what is ‘long’ in comparison to what is ‘short’, similarly to the meaning of ‘clean’ being defined in references to the meaning of ‘dirty’ and so forth.

SRT refers to the notion of themata to explicate how antinomies that have spurred public discussions, debates or conflicts can become thematized (Marková 2000), evolving from basic dichotomies existing in a dormant state in the collective memory to themata.

In this view, it is possible to posit that current discussions on WHR ranking of Finland as happiest country in the world on the one hand, and the high levels of depression among general Finnish population on the other hand, could also contribute to broader debates and arguments around the taxonomy of happiness/unhappiness, influencing the way laypeople make sense of these concepts.

### Research questions

With the exception of a limited number of studies (e.g., Delle Fave et al. 2016; Rodríguez-Araneda 2013; Shin et al. 2018), there is a paucity of empirical studies approaching everyday understandings of happiness, and even fewer studies (e.g. Uchida and Kitayama 2009) have encompassed everyday understandings of unhappiness.

We propose that mixed methods extending beyond the scaled instruments traditionally employed in well-being research are required to capture shared common-sense conceptions of happiness in different contexts, settings and social groups.

In the current study, we employ word associations, a technique frequently used for the analysis of social representations of topical issues (e.g., Wagner et al. 1996; Moloney et al. 2015) with the aim of exploring what kinds of everyday ideas young (16–18-year-old) and adult (29–34-year-old) women in Finland have about happiness and unhappiness, and how these ideas relate to their self-reported happiness levels. In addition, we also look at the participants’ beliefs on how (and if) one’s level of happiness can be improved.

## Method

### Participants

Altogether 409 participants from the Helsinki region participated in this study: 220 female upper-secondary school students (16–18 years old) and 189 adult women (29–34 years old). The two age groups were recruited respectively in 2016 and 2018.

The 16–18-year-old group was recruited from nine upper-secondary schools located in the Helsinki region. We selected upper-secondary schools located in various districts of the urban capital region and specializing in different subjects. In Finland, students can choose themselves which upper-secondary school to apply for, regardless of their domicile. Moreover, many upper-secondary schools allow students to focus on certain areas defined by a national program for school specialization (including, for example, arts, sports, music and science).

The first author contacted the principals of the selected schools and booked an appointment with the ones that were interested in participating in the study. No compensation was offered and the participants were recruited on a volunteer basis. The participants were then given approximately 45 min to fill in a paper version of the questionnaire.

The 29–34-year-old group was recruited through a link to an e-form version of the questionnaire share on Facebook. As radically different as it may seem to recruit a second group of participants online when the first one was given the option to fill in a paper version, we argue that it would be virtually impossible to replicate the data collection system implemented in upper-secondary schools with a group of adults while simultaneously retaining the same variations in terms of geographical location and area of expertise of the participants. The link to the e-form was shared on various closed Facebook groups. These included groups for local communities in different areas of the Helsinki region (e.g., Itäkeskus- itästadilaista laiffii, “Itäkeskus and Eastern Helsinki area Life”; Puskaradio, “Grapevine/gossip”), parents’ groups linked to different neighborhoods (e.g., Jätkäsaaren ja Ruoholahden vanhemmat, “Parents of Jätkäsaari and Ruoholahti”), and women-specific groups (e.g., Naistenhuone, “Women’s room”). Since most closed Facebook groups are intended for users fulfilling specific requirements (e.g., a specific domicile in the case of local community groups), the primary author contacted the admin of each group through Facebook’s private message function, in order to introduce the general aim of the research and ask for permission to share the link to the e-form in the group. Once we obtained permission, we made posts asking those members of the groups fulfilling the participation criteria to fill in the e-form. After participants ticked the box indicating that they had read and understood the informed consent form, they had access to the online questionnaire.

### Material

We collected the data through a word association task located at the start of a longer questionnaire designed by the first and third authors. The stimulus words, as well as the rest of the questionnaire, invitation, and informed consent form, were presented in Finnish. Participants were asked to write down, without thinking too much, the first five words or ideas that first came to their mind when thinking of the stimulus word *onnellisuus* (‘happiness’). Subsequently, the task asked participants to share what, in their view, represents *onnellisuuden vastakohta* (‘opposite of happiness’), by writing down the first five words that first came to their mind when thinking of that. As Finnish language does not have a proper equivalent of the English term ‘unhappiness,’ we considered that forcing on the participants an assigned term to define the concept (for example, *suru*, ‘grief’) would have been antithetical to the basic premises and aims of our study. However, we would like the reader to notice that in the present paper, for the sake of consistency with previous studies and fluency in English language, we use the term ‘unhappiness’ to refer to the ‘opposite of happiness’.

Word associations comprise a technique frequently used for the analysis of social representations of topical issues (Dany et al. 2015). We had a twofold rationale for choosing this technique. First, in line with our theoretical approach, this method is particularly apt for mapping out the structural aspects of socially shared thinking, instead of merely exploring individual cognitive structures (Mäkiniemi et al. 2011). Secondly, the combination of spontaneity, characterizing the nature of the task, and the possibility to write the responses may diminish the social desirability effect and similar qualms related to the reliability of answers to questions about the elusive subject of happiness (Brulé and Veenhoven 2017).

After completing the word associations task, participants were asked to rate the level of their own current happiness with a scale item: “*On a scale of 1–10, how happy are you?*” This was followed by the open-ended question: “ *If your answer to the previous question wasn’t 10, can you think of what would have to change in your life so that you could answer ‘10’?*”

The rest of the questionnaire contained items related to possible gender-specific understandings of happiness, which were not used in the present analysis, and various demographic questions. The data that support the findings of this study are available on request from the corresponding author.

### Analytical Procedure

The associations generated were prepared for statistical analysis by the first author following the guidelines set forth by Wagner (1997, p. 7) of treating the words as much as possible “as they are,” rather than categorizing them according to a scheme imposed by the researchers. When dealing with word associations “as they are,” the associations generated undergo an initial “cleaning” process, which consists of a systematic synonym reduction. Before the word associations could be cleaned and homogenized, we inspected the data for errors, such as typing mistakes, and compiled two lists of the words in alphabetical order, one for the associations with ‘happiness’ and one for ‘unhappiness.’ The associations produced were typically single words (e.g., *vapaus*, ‘freedom’) and, more rarely, short sentences (e.g., *vapaus tehdä mitä vain*, ‘freedom to do whatever’) or words in pairs (e.g., *hymy/nauru*, ‘smile/laughter’). Whenever possible, short sentence were reduced to the most similar single word (e.g., ‘freedom to do whatever’ would be merged with <freedom>) and words appearing in pairs would be merged with single words, taking into account the first word mentioned by the participant (e.g., ‘smile/laughter’ would be merged with <smile>). After the edited lists were organized in both alphabetic and frequency order, we adopted a twofold criterion as guidance in the synonym reduction process: the criterion of a common root word (Moloney et al. 2015) and the homogenization of semantically equivalent words (Wagner 1997). The common root word technique refers to grouping together verbs, nouns or adjectives stemming from the same basic part of a word, without taking suffixes and singular/plural forms into account. For example, the terms *lapsi* (‘child’), *omat lapset* (‘own children’) and *lapset* (‘children’) were categorized under the word category <children>, which was the most frequent variation of the term. Similarly, the words *ilo* (‘joy’) and *iloinen* (‘joyful’) were categorized under <joy>, which again was the most dominant variation of term. This phase was particularly relevant, considering that Finnish is not an Indo-European language but rather a Finno-Ugric language belonging to the Uralic language family. As such, Finnish is an agglutinative language, meaning that words are composed of a string of morphemes representing single grammatical categories.

After completing this first selection procedure, the associations were also homogenized according to semantically equivalent terms. For example, *rahaongelmat* (‘money problems’) and *taloudelliset huolet* (lit. ‘economy-related worries,’ or ‘economic worries’) were grouped under <money/economic problems>, since there existed a rather wide range of heterogeneous terms used to express the same concept. In this phase, the first author cooperated closely with the third and fourth authors, who are native Finnish speakers, to find a common agreement regarding which minor semantic variations could be retained in order to preserve the integrity of the data.

Finally, the newly compiled lists of associations with the concepts “happiness” and “unhappiness” were separately ranked by frequency in each age group. The two resulting lists were compared between the two age groups and homogenized as follows: for each of the four lists, we selected the 20 words with the highest frequency. After that, we looked at the differences in the top 20 “happiness” words between the two age groups. If a word figuring among the “top 20 words” for “happiness” in the younger group list occurred at least five times in the “happiness” list compiled for the older age group, then the term was selected as a final common shared category. The same rationale was used to inspect the lists in the reverse order (older age group to younger age group), and the entire procedure was then repeated for the two lists of words associated with “unhappiness”. Terms that had a high frequency (*N* > 10) in the list of one of the age groups but low (or zero) frequency in the other age group were included as well.

## Results

### Data structure

To recap, the data consist of two sets of word associations with stimulus words ‘happiness’ and ‘unhappiness’ a self-rated happiness scale from 0 to 10 and an additional item elaborating on the participants’ understandings of how to possibly reach 10 on such scale. We will start by presenting the results of the word associations, which were subject to the analytical procedure described above. We obtained four separate lists of words: 1) associations with ‘happiness’ (16–18 years old; 2) associations with ‘happiness’ (29–34 years old.); 3) associations with ‘unhappiness’ (16–18 years old); and 4) associations with ‘unhappiness’ (29–34 years old). The associations making up the four lists and their respective frequencies are reported in Table 1.

**Table 1:** Frequency of word associations with stimuli terms ‘Happiness’ and ‘Opposite of Happiness’

Associations with ‘Happiness’	*f* (16–18 year olds)	*f* (29–34 year olds)	Associations with ‘Opposite of happiness’	*f* (16–18 year olds)	*f* (29–34 year olds)
*Family*	140	95	*Loneliness*	93	72
*Friends*	141	44	*Grief*	55	43
*Love*	53	63	*Anxiety*	38	47
*Health*	50	53	*Depression*	57	21
*Joy*	51	27	*Sickness*	40	32
*Calm*	16	40	*Crying*	40	13
*Smile*	33	18	*Stress*	33	17
*Freedom*	22	30	*Fear*	17	25
*Food*	46	0	*Death*	26	11
*Home*	13	28	*Hate*	18	13
*Romantic Relationships*	17	25	*Quarrels*	21	7
*Security*	0	35	*Poverty*	23	10
*Money/finance*	24	28	*Low spirits*	16	12
*Balance*	5	31	*Worries*	8	20
*Laughter*	25	5	*Fatigue*	18	8
*Sun*	23	0	*Pain*	9	12
*Warmth*	11	14	*Financial problems*	9	15
*Contentment*	10	14	*Darkness*	11	9
*Nature*	5	17	*Loss*	11	9
*Work/School*	9	13	*Boredom*	10	10
*Sport*	19	0	*Failure*	11	5
*Positivity*	18	0	*Feeling bad*	16	0
*Traveling*	13	5	*No perspective*	0	12
*Good feeling*	8	11	*Insecurity about oneself*	9	0
*Music*	18	0	*Insecurity*	0	11
*Summer*	15	0	*Uncertainty*	0	10
*Children*	0	18	*Hopelessness*	0	10
*Hobbies*	17	5			
*Close people*	28	9			

We used the data reported in Table 1 to perform CA (Greenacre 1993) to graphically illustrate what words had consistently co-occurred in the answers of the same participants. For this purpose, we organized each of the four word lists into four co-occurrence matrices, where the diagonal represented the absolute frequency of each word.

**Fig. 1:**
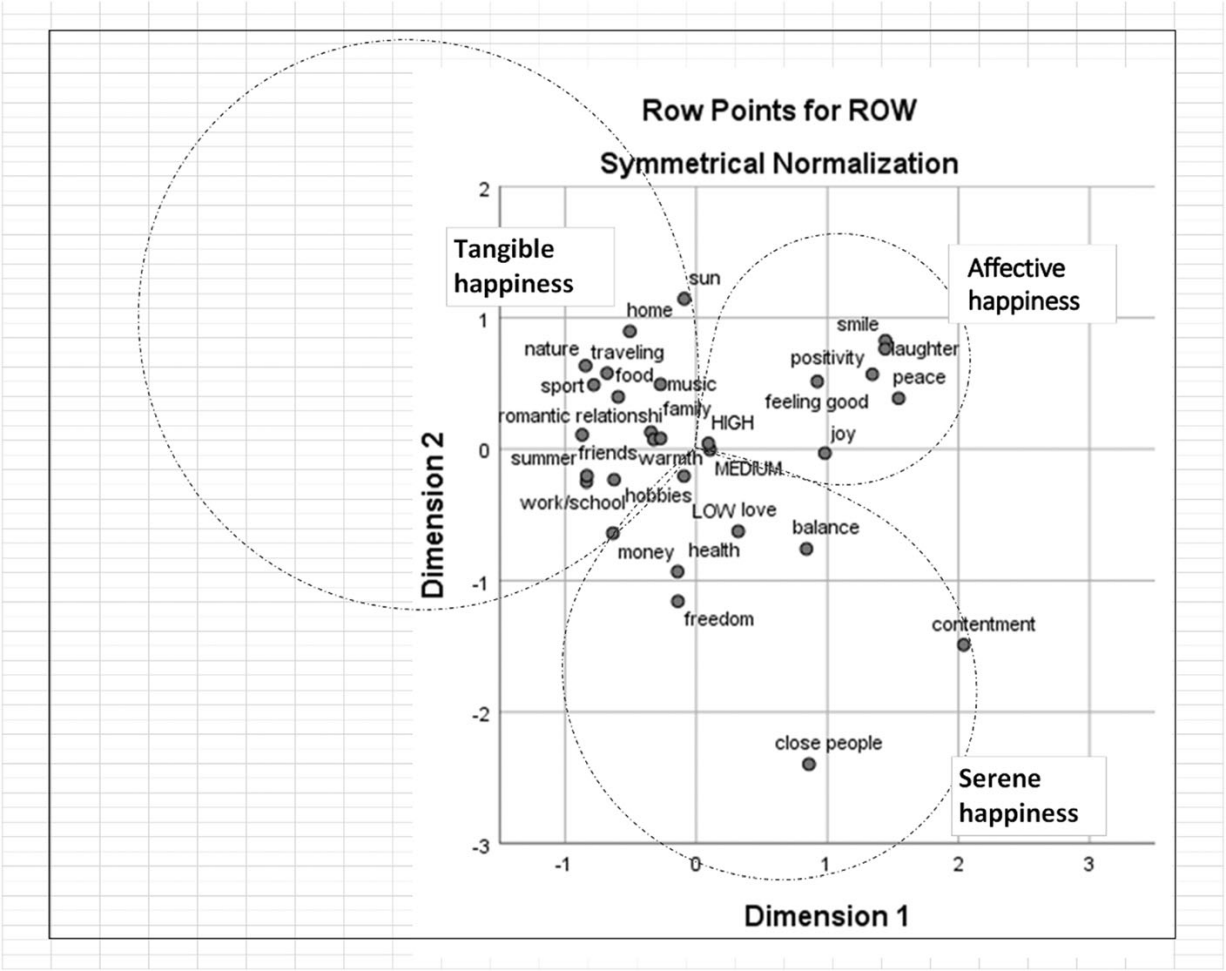
Correspondence analysis of word associations produced by 16–18 years old women with the term ‘happiness’

**Fig. 2:**
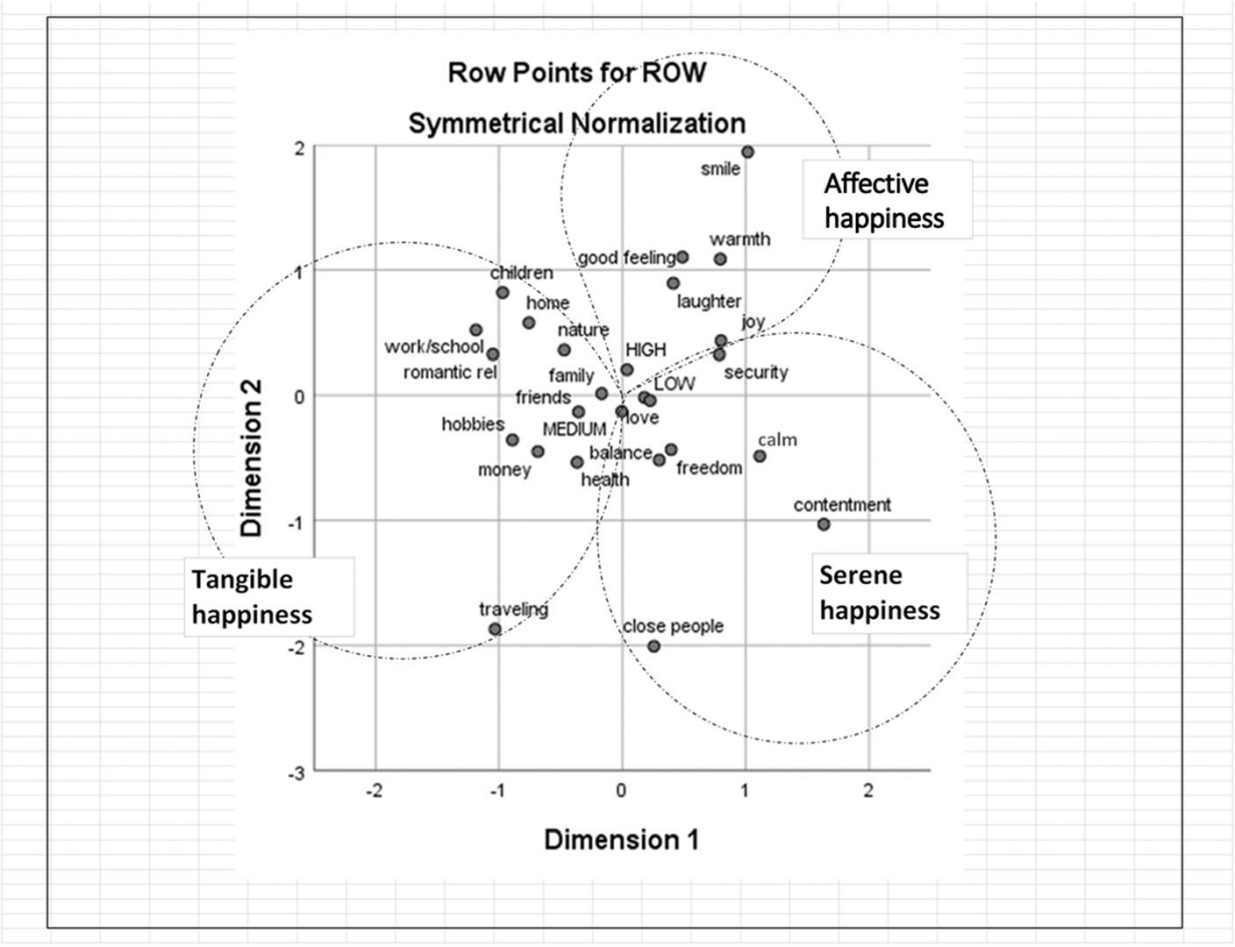
Correspondence analysis of word associations produced by 29–34 years old women with the term ‘happiness’

**Fig. 3:**
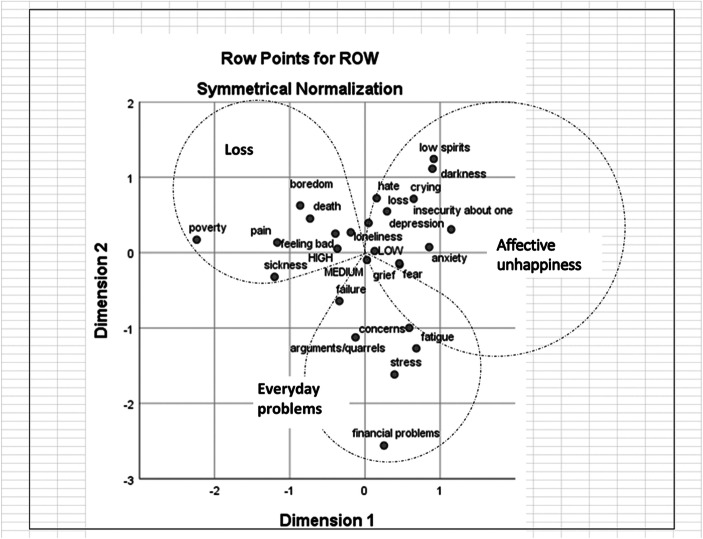
Correspondence analysis of word associations produced by 16–18 years old women with the term ‘unhappiness’

**Fig. 4:**
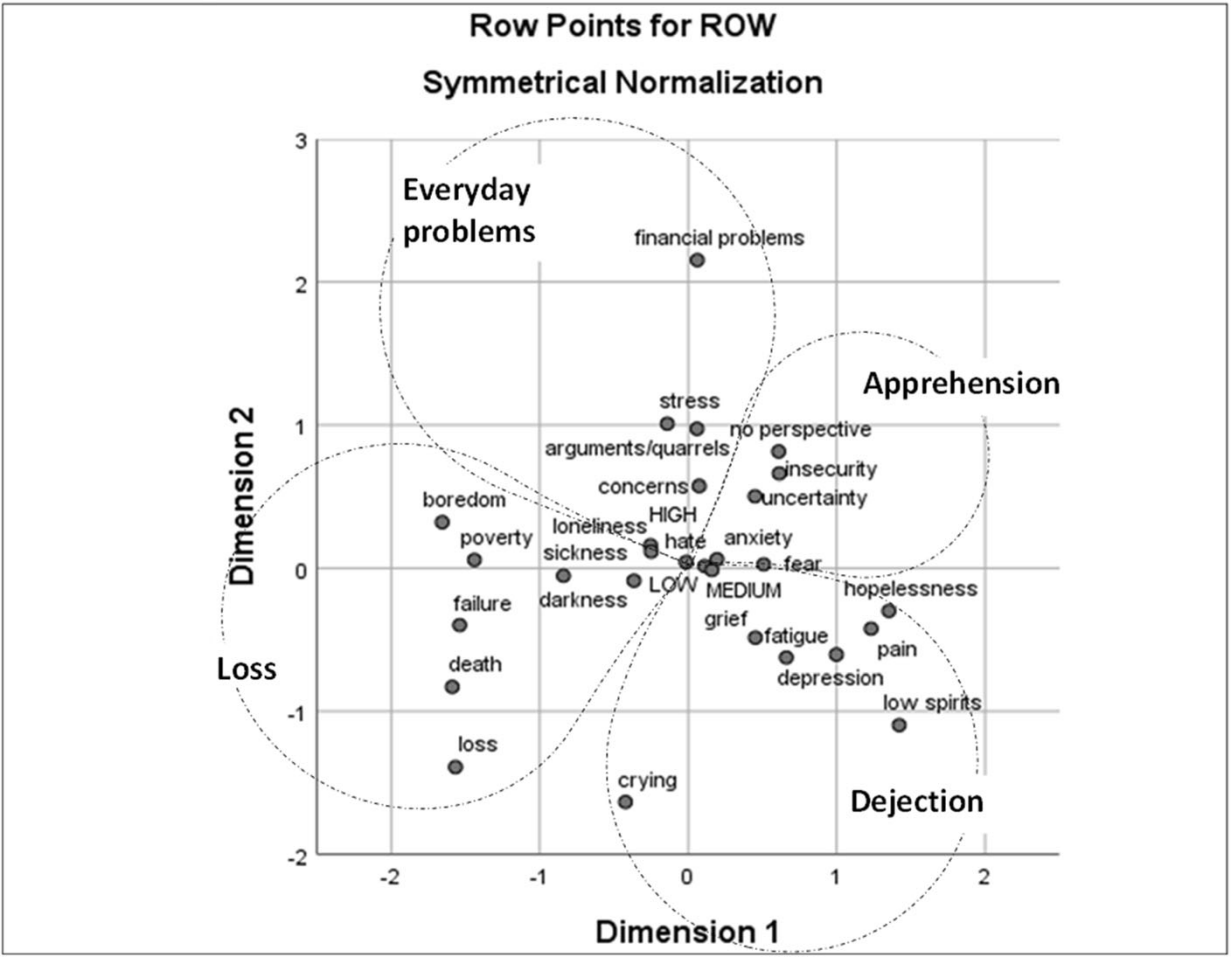
Correspondence analysis of word associations produced by 29–34 years old women with the term ‘unhappiness’

We used SPSS Statistics 25 to run the CA and a subsequent Hierarchical Cluster Analysis (HCA) over Dimensions 1 and 2, which emerged from the CA. When calculating the HCA, we considered the cos(θ) (*cosine distance*) between the word associations, as this is the most indicated metric for measuring variable distances in exploratory settings where the vectors’ magnitude of text data is not relevant. We calculated 3 to 6 clustering solution dendrograms for each of the four word lists and mapped them onto the CA plots (Figs. 1, 2, 3 and 4). In the following paragraphs, we elaborate on the relationship between the words contained in each cluster, which we refer to as “semantic repertoires.”

#### Semantic Repertoires of Happiness

In the figures below the dotted lines show the extent of the semantic repertoires based on the dendrograms produced with Hierarchical cluster analysis. We named the three cluster solutions (semantic repertoires) that gave the most meaningful/interpretable results with happiness-related terms: *Tangible happiness*, *Affective happiness* and *Serene happiness*, based on the commonalities found in the words.

The first semantic repertoire, *Tangible happiness*, contains words expressing concrete aspects of happiness, embodied in the form of people (e.g., family, friends, children), material assets (e.g., home, money), activities (e.g., traveling, hobbies) and places (e.g., work, school). *Tangible happiness* is the repertoire that contains both the highest number of associations and the highest number of differences between age groups. More specifically, the words ‘music,’ ‘sun,’ ‘summer,’ ‘sports’ and ‘warmth’ make up a big part of *Tangible happiness* for younger women, whereas older women do not mention these terms, and include the term ‘children’ in this repertoire.

The repertoire *Affective happiness* contains words that refer to emotional states (e.g., joy) as well as physiological (e.g., warmth) and expressive (e.g., smiling, laughter) responses commonly associated with happiness. The words contained in this repertoire are nearly identical between the age groups, with the exception of the word ‘positivity,’ being mentioned only by younger women and ‘security’ and ‘warmth’ being mentioned only by older women.

Finally, the words contained in the repertoire *Serene happiness* represent long-term desired life standards, ranging from more abstract constructs (e.g., freedom, contentment) to more concrete expressions of states of being (e.g., health, balance). Younger women include also the term ‘health’ in this repertoire, whereas older women include the term ‘calm.’

#### Semantic Repertoires of Unhappiness

The repertoires that emerged when exploring the associations made with “unhappiness” were less structured than their “happiness” counterparts. Moreover, there are clear structural differences between the age groups.

For the younger women it was possible to identify three repertoires, which we have named *Loss*, *Everyday problems* and *Affective unhappiness*. For the older women, the representations of unhappiness appear much more fragmented and heterogeneous.

Both age groups share the repertoire *Loss*, which includes associations with the loss of something positive: sickness is a loss of health, poverty is a loss of wealth, boredom a loss of meaningful/interesting activities and so on. Therefore, this repertoire refers to conditions of deprivation, where the things that are missed create a state of misery.

The repertoire *Everyday problems*, like the first repertoire, is shared between the two age groups, and it reflects a more mundane array of issues which can be considered more transitory and part of everyday challenges.

Younger women present a clear third repertoire, *Affective unhappiness*, which contains terms referring to a “general idea” of unhappiness, including an array of negative emotional states (e.g., low spirits, grief, anxiety) and related responses (e.g., crying). The associations produced by the older women generated a third and a fourth repertoire, which we named respectively *Dejection* and *Apprehension*. As suggested by the term, *Dejection* refers to dejection-based feelings, characterized by a state of depression or tiredness. On the other hand, *Apprehension* includes associations related to the state of alertness induced by fear, anxiety, stress, etc.

#### Self-Rated Levels of Happiness

The self-rated levels of happiness on a scale from 0 to 10 were divided into three categories as follows: low (0–7); medium (8) and high (9–10). These were added to the co-occurrence tables as passive variables when calculating the CA. Figures 1, 2, 3 and 4 show that the self-rated levels of happiness are not discriminatory in terms of what words are most likely to co-occur in the responses of the same participants. The result is virtually the same for words associated with both stimuli words ‘happiness’ and ‘unhappiness’ and for both age groups.

The answers to the open-ended question item “*If your answer to the previous question wasn’t 10, can you think of what would have to change in your life so that you could answer ‘10’?*” were analyzed by using qualitative data-driven content analysis. We adopted an inductive analytical approach, beginning with careful readings and re-readings of the material followed by generating data-driven codes. Finally, we searched for broader themes under which to categorize the codes generated while going back and forth between the code list and the original text. The codes and the related themes that emerged are presented in Table 2.

**Table 2:** Answers by participants (*N* = 370) to the open-ended question item “*If your answer to the previous question wasn’t 10, can you think of what would have to change in your life so that you could answer ‘10’?*”, Self Confidence

Internal (Self) *N* = 67	External (Circumstances) *N* = 279	10 is “Unattainable” *N* = 24
Self-confidence	Money	Always room for improvement
Self-discipline/Energy	Relationships/Loneliness	Happiness is fleeting
Positivity	Health	
	Stress/No time	
	More interesting/meaningful activities	

Our analysis suggested three recurring answer types: happiness can be improved through external changes, internal changes, or not at all because complete or permanent happiness does not exist. We further elaborate on the significance of these answers in the discussion section below.

## Discussion

### Varieties of (un)happiness

The main objective of this study was to explore what kind of everyday notions young (16–18-year-old) and adult (29–34-year-old) women in Finland have about happiness and unhappiness. Additionally, we sought to explore how these understandings related to the participants’ self-reported happiness levels and what they thought about reaching the highest possible happiness level.

The findings of the present study highlighted that the social representations of happiness in both age groups are structured in a very similar way. Three clear semantic repertoires emerged from the HCA: *Tangible happiness*, *Affective happiness* and *Serene happiness*. Although the structure and contents of these repertoires greatly overlap between the two age groups, there are also some interesting differences. For example, in *Tangible happiness*, we noted that younger women mention words that are intuitively closer to “enjoyment” (e.g., music, sun, summer, sports, and warmth). On the other hand, when we look at the same semantic repertoire in the data of the older women, these “enjoyment” words are missing, while the word ‘children,’ which did not appear among younger women, clearly has an important focus. This is perhaps not surprising, considering that according to Statistics Finland, 30 is the average age for women in Finland to get their first baby. These variations in the concrete elements associated with happiness between the two age groups corroborate previous Finnish studies; for example, Pessi (2008) found that *human relationships*, *health* and *secure income* are important elements for Finnish people in general, but that *hobbies* hold a prominent position among young people in particular. All considered, we suggest that *Tangible happiness* represents a way of defining happiness by looking at its concrete (or, as the name suggests, tangible) antecedents. In other words, this repertoire resonates with the question *What makes people happy?*

The repertoire of *Affective happiness*, which is nearly identical between the age groups, includes both notions of positive affects and the way positive feelings are externalized and made concrete. The associations contained in this repertoire align with the “folk prototype” of happiness proposed by Kovecsez (Kövecses 2008), characterized by a “high degree of noticeability [and] dominated by highly noticeable behavioral, physiological, and expressive responses” (p. 139), as well as with an understanding of happiness that is close to the concept of joy. Presuming that *Affective happiness* reproduces social conventions of how happiness should be expressed, shared and communicated, we suggest that this second semantic repertoire constitutes what in social representations theory terms would be called the *objectification of happiness*, namely, the way the abstract nature of happiness is transformed into a more concrete image. Simply put, we suggest that *Affective happiness* informs what happiness is expected to “look like.” Interestingly, what we are expressing in social representations terms resonates with empirical studies that, stemming from very different epistemological assumptions, have focused on the way emotions like happiness are made visible through facial expressions (e.g., Hager and Ekman 1979). Almost all empirical research conducted in this area has shown that people consistently connect happiness with the same facial expression (i.e., smiling), making happiness one of the most easily and unambiguously recognizable emotions (Hager and Ekman 1979). We suggest that such notions regarding the way happiness is expressed, which have stemmed from purely cognitive traditions, have penetrated the way we think and discuss the question *what does happiness look like?* This suggestion does not appear too far-fetched when one considers the incontestable role that emojis, especially “smileys,” have acquired in modulating our everyday communications, serving the important function of “providing visual imagery to the writing” (Danesi 2016, p.11). As our informants have noted,^1^*Affective happiness*, being concerned with what happiness looks like, is more easily rendered visible and recognized in others rather than in ourselves. In other words, we might not resort to the same image of happiness when gauging our own and other people’s happiness, simply because popularized images of what happiness is supposed to look like (i.e., smiling, laughing, joy) are more noticeable when looking at someone else from the outside.

Finally, the repertoire *Serene happiness* can be intuitively connected to more stable connotations of happiness, which have a longer, albeit less visible, lifespan compared to *Affective happiness*. Instead of fleeting emotions, the third semantic repertoire hints at a more harmonic vision of happiness, closer to the idea of fulfilment and satisfaction. Interestingly, younger women include ‘health’ in this third repertoire, whereas for older women, who have probably had a few more years to encounter some health-related problems (either directly or indirectly), ‘health’ is instead part of the *Tangible happiness* repertoire.

As regards to ‘unhappiness’, the various repertoires that emerged when were less homogenous than their “happiness” counterparts. In terms of “unhappiness,” the two groups share only two semantic repertoires: *Loss* and *Everyday problems*. A distinct third semantic repertoire, which we named *Affective unhappiness*, emerged for the younger women; it resonated—and, to a certain extent, mirrored—with the content of the semantic repertoire *Affective happiness.* Thus, we suggest that *Affective unhappiness* might be the way younger women turn the vagueness elicited by “unhappiness”^2^ into a more concrete image (e.g., crying, low spirits, anxiety), corresponding to the objectification of happiness.

Older women’s associations with “unhappiness” are further differentiated into a more complex structure, which includes two additional semantic repertoires: *Dejection* and *Apprehension*. When thinking or discussing about mental disorders, there is a chance that most laypeople would name something like depression and/or anxiety. Depressive and anxiety disorders are in fact the two main categories of mental disorders, and they are considered “common” because they are highly prevalent in the general population (WHO 2017). We deem it possible that such scientific notions on mental disorders have trickled down to discussions among the general public and contributed to shaping two different facets of unhappiness. On the one hand, as shown in the semantic repertoire *Dejection*, unhappiness is characterized by a range of negative affects and states that indicate an overall *slowing down* motion (e.g., low spirits, depression, fatigue). On the other hand the semantic repertoire *Apprehension*, which locates unhappiness around notions supposedly derived from the category of anxiety disorders, is characterized by words that suggest overall *restlessness* (e.g., insecurity, fear, anxiety).

With this study, unlike what is done in the majority of happiness studies, we wanted to see what kind of semantic repertoires would be produced when we gave participants the freedom to come up with their own meaning(s) for happiness/unhappiness. As mentioned in the introduction, little is known about laypeople’s understanding of happiness. So far, the relatively limited number of studies which have focused on common sense notions of happiness appears to be heterogeneous in respect to the phrasing of the questions employed to explore definitions of happiness, which can range between its antecedents (‘*what makes you happy?*’) and the meaning of happiness as a concept (‘*what is happiness for you?*’). Overall, these studies have highlighted cultural differences as well as similarities in conceptualizations of happiness, identifying social relationships as one of the most universally shared component of happiness (Delle Fave et al. 2016). Our findings suggest that in laypeople’s minds happiness can be understood, at the same time, as both the factors that lead to happiness (repertoire Tangible happiness) and as a state or experience which can be either fleeting (repertoire Affective happiness) or long-lasting (repertoire Serene happiness). In addition, in line with Uchida and Kitayama (2009) our results show that the repertoires associated with unhappiness are not a faithful reflection of the repertoires associated but with happiness, but rather present greater complexity, which seems to increase with age. In SRT terms, these results are in line with the idea that not all oppositional pairs are perfectly symmetrical in their symbolic value: for example, in the left/right dichotomy, the value culturally placed on the left hand differs at a symbolic level from the value attributed to the right hand (Wagner 2020). Similarly, although common sense of notions of happiness can be partly shaped by common sense notions of unhappiness, the two notions do not represent two mutually exclusive opposites on a continuous dimension. This cognitive asymmetry also exists between the concepts of war and peace (Wagner et al. 1996). That is, they are not exactly what Marková (2000) described as exclusive cognitive poles of one theme. The way these two concepts are made sense of in reference to each other does indicate significant cultural variations.

### Between Happiness and Unhappiness: Is there a “Way Up”?

The qualitative content analysis of participants’ answers to the questionnaire item asking what would have to change in their life for them to answer 10 when asked how happy they feel on scale on a scale from 1 to 10 produced three main themes, which were shown in Table 2. The first theme, *External-circumstances*, is the most dominant view on how happiness can be improved. The most reoccurring sub-themes included having “more” of something: more money, time, friends, better health. To a certain extent, these sub-themes are in line with the results obtained by Ojanen (2008), as well as with the semantic repertoire we presented as *Tangible happiness*, which included concrete and specific assets associated with happiness.

The second theme that emerged from the data, *Internal-self*, suggests instead that the highest level of happiness can be reached by changing oneself rather than hoping for a change in external factors. The sub-themes belonging to this group focused around changing one’s attitude and thoughts toward the positive, improving on different fronts while at the same time being more confident and acceptant toward oneself. To better understand the difference between the first and second theme, let us take “positive relationships with loved ones” (Ojanen, p. 46) leading to greater happiness as an example. While the answers in the theme *External-circumstance* would have mentioned this aspect simply by indicating “more friends” as a solution, answers in the theme *Internal-self* looked like the following instead:

I don’t know [what would have to change so I could reach 10 on a 1–10 happiness scale]. Maybe nothing? But I could learn to find comfort in myself in the (rare) moments in which I feel lonely: learning to be alone.

Interestingly, the third and final theme identified through our analysis, named *10 is unattainable*, includes answers which, unlike the previous themes, challenge the setting proposed by the researchers, arguing that “10” is a bogus number on the happiness scale considered, which is supposed to be there only to remind us that *complete happiness* is elusive. The reasons given (indicated through the sub-themes) include the fleeting nature of happiness, which, by default, prevents us from answering “10” at any given time, or, on the other hand, the tendency to believe that there is always “something better around the corner,” as indicated in the following extract:

It’s difficult to ever say 10, when you never really know [even when you are happy] how much happier you could still be.

To sum up, while most people seem to attribute the possibility of becoming happier to changes which can be either external or internal, a minority thinks that complete happiness is unattainable. While there is empirical evidence showing that people who believe they can influence their level of happiness are in fact happier than those who do not believe they can (Ojanen 2008), we think that this might not *always* be the case. Relying on internal rather than external changes could also add to the pressure of being responsible for one’s own ability (or inability) to implement such changes. In contrast, considering the highest possible level of happiness as “unattainable,” and thus aligning with representations of happiness as an “elusive” state rather than a goal to reach, might in the end translate into higher levels of self-assessed happiness. This vision of happiness resonates in the famous folk wisdom quote “Happiness is a journey, not a destination,” often seen printed on cards, coffee mugs, fridge magnets or shared on the internet.

#### Limitations and Future Directions

The present study presents a number of limitations. First, although justified because of the higher incidents of depression among women in the Finnish general population, our decision to focus on the way women understand happiness and unhappiness in Finland would have yielded clearer results if compared to social representations of happiness/unhappiness among Finnish men.

Secondly, although we had reasons to select two specific age groups based on *Generations Y* and *Z* and on ages 18–16 and 29–30 being two relevant life periods for Finnish women, we would like to acknowledge that there are other age groups (for example, seniors), which in the future should be included in such studies, as they might be likely to produce different understandings of happiness.

Finally, we would like to invite the reader to note that CA is a thoroughly descriptive technique; thus, results showing the relationship within words, as well as the relationships between words and self-reported happiness levels, are not based on inference statistics but on an exploratory design. Future studies could be more creative in coming up with mixed-method designs to move more fluidly between qualitative, non-parametric and parametric approaches.

### Conclusion

In spite of the limitations discussed above, we believe our findings contribute to a more nuanced understanding of the way laypeople make sense of the abstract concept of happiness.

First, our findings suggest that happiness is understood in three similar ways among both Finnish young and adult women: *Tangible happiness* (what makes us happy), *Affective happiness* (what happiness looks like), and *Serene happiness* (what long-lasting happiness is). If, as our findings suggest, the objectification of happiness relies on a common understanding of what happiness is supposed to *look like* (*Affective happiness*), we can understand why Finland being reported as the “happiest country in the world” can potentially clash with the discourse of laypeople around Finns being emotionally introverted people. We suggest that this clash constitutes a possible explanation why Finland’s position in the WHR was received at the same time as a “joke” and as something which “makes sense,” as the young woman cited at the beginning of this paper pointed out.

Secondly, our findings suggest that the social representations of “happiness” shared by Finnish women are not completely antithetical with their representations of “unhappiness”; more specifically, those of “unhappiness” are more complex and heterogeneous than their “happiness” counterparts. Thus, Tolstoy’s words “All happy families are alike; each unhappy family is unhappy in its own way”—reflecting the idea that happy people share a common set of attributes which lead to happiness while unhappiness yields greater complexity and diversification—seem to hold a kernel of truth when looking at our results. While there is an *Affective happiness* which can be mirrored in the objectification of “unhappiness” (*Affective unhappiness*), at least among younger women, it also seems that there are other semantic repertoires or understandings of happiness which do not find such a perfect semantic match in their unhappiness counterpart. Thus, to answer the question posed in the title of the present paper, happiness and unhappiness do not seem to map onto the figurative heads or tails of the same coin, but rather seem to constitute two separate dimensions, or two different “currencies,” constituting two separate sets of meanings. This finding could be considered a plausible explanation for why the same country could score high in the World Happiness Report and, at the same time, have high depression rates. Based on our results, we argue that releasing survey results on life satisfaction/quality of life using the term ‘happiness’ (such as the WHR), on the one hand, and the assumption that depression rates can be regarded as symmetrical or a continuous opposite of happiness, on the other hand, are not in line with everyday understandings of happiness and unhappiness.

Finally, while self-rated levels of happiness do not seem to elicit associations to either happiness or unhappiness, we propose that there are different understandings of how (and if) the highest levels of happiness can be reached, namely, a change of external circumstances, a change within the self or, finally, acknowledgement of the idea that reaching the highest degree of happiness at any given time is simply impossible.

In conclusion, our study offers a new means to study happiness, starting with the way in which laypeople understand the concept and extending to how they understand its opposite.

As lay discourse is understood to be culturally and historically situated, we believe that taking into account emic approaches in cross-cultural studies on well-being could have a number of practical implications.

First, delving deeper into cultural differences and similarities of the notion of happiness (and unhappiness) could provide tools for developing new models and approaches to empirically investigate happiness as a universal yet cultural-specific concept. From a methodological perspective, most scaled instruments used in surveys measuring happiness, are in fact designed in academic contexts and directly applied to participants without consideration for local processes of sense-making of happiness and unhappiness, and without critical awareness (Christopher et al. 2014) of the academic grounding underpinning the design of the surveys.

Secondly, in line with Delle Fave et al. (2016) we believe it is crucial to acknowledge that cross-cultural studies deal with an increasingly multicultural scenario, where different cultural traditions and groups co-exists in the same country. Unpacking everyday understanding of happiness and unhappiness could represent an invaluable asset for policies aimed at increasing the well-being and flourishing of societies in their complex diversity.

## Data Availability

The data that support the findings of this study are currently in the process of being archived at the Finnish Social Science Data Archive (FSD) and will be accessible by registered users for both research and teaching purposes. Link to the FSD: https://www.fsd.tuni.fi/en/

## Footnotes

1 As the first age group considered in this study is not close to the generations that the authors belong to, we conducted a discussion with three 18-year-old female high-school students in Finland to get better acquainted with the linguistic repertoire commonly used when talking about happiness/unhappiness and to corroborate our interpretations of the statistical results.

2 Our informants (see footnote 1) could not agree on the noun indicating the exact opposite of happiness in the Finnish language. They instead settled for the term *onneton*, which corresponds to the English adjective ‘unhappy.’
